# Dual sagittal-guided coarse-to-fine rib segmentation and numbering in chest CT

**DOI:** 10.1007/s11517-026-03576-2

**Published:** 2026-05-22

**Authors:** Seonghyeon Ko, Duc-Tai Le, Junghyun Bum, Hyunseung Choo

**Affiliations:** 1 Department of AI Systems Engineering, Sungkyunkwan University, Suwon, Korea; 2 Department of Electrical and Computer Engineering, Sungkyunkwan University, Suwon, Korea; 3 College of Computing and Informatics, Sungkyunkwan University, Suwon, Korea

**Keywords:** Coarse-to-fine rib segmentation, Rib numbering, Deep learning, Chest computed tomography

## Abstract

**Abstract:**

Rib identification in chest CT is time-consuming and labor-intensive, as clinicians repetitively examine hundreds of CT slices to pinpoint a certain rib on a random slice. Computer-aided rib numbering tools exist, however, their performance hinges on precise rib segmentation which is challenged by missing ribs or oversegmentation of adjacent bone structures. We propose Dual sagittal-guided Coarse-to-fine Rib Segmentation (DCRS), a lightweight and practical 3D rib segmentation approach that coarsely captures the entire rib structure within 2 sagittal slices and finely constructs 3D ribs. DCRS operates in three stages: (1) Region of Non-Interest (RONI) removal, a preprocessing stage which excludes the soft tissues, spine, sternum, and transverse processes to isolate ribs; (2) a lightweight U-Net applied to only two representative sagittal slices for obtaining coarse sagittal ribs; and (3) Six-Neighborhood Outward Flood-Filling (SNOFF), a fine 3D rib construction that expands sagittal rib predictions into full 3D ribs. This coarse-to-fine approach ensures a complete 12-pair rib mask while decreasing the complexity and computational cost compared to coarse slice, patch, or point-cloud-wise rib segmentation methods. The training and testing of the proposed DCRS utilize a public rib segmentation dataset. DCRS achieves a Dice score of 92.83% and IoU of 88.21%, exceeding prior state-of-the-art by 3.1% and 4.1%, respectively. DCRS enables fast and reliable rib numbering, increasing throughput by 44% compared to the prior state-of-the-art. Rib numbering takes only 0.5% additional time relative to standard CT, highlighting its clinical practicality.

**Graphical abstract:**

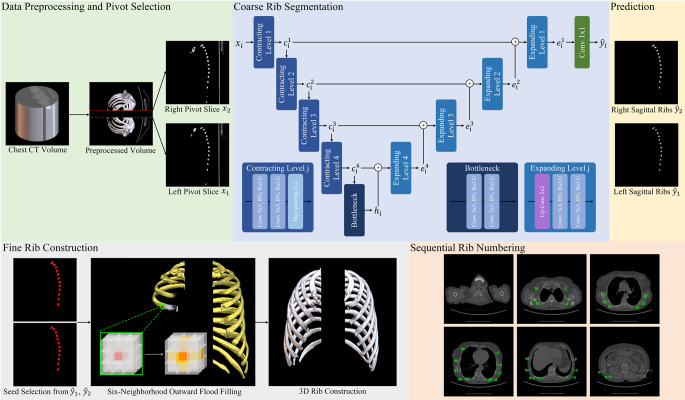

## Introduction

**Fig. 1:**
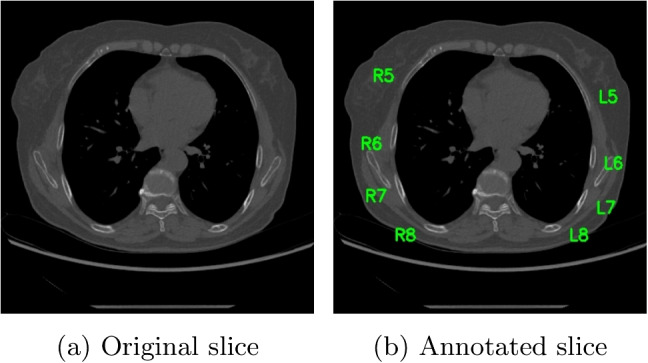
An axial chest CT slice and slice with rib annotations. The original slice requires repetitive investigation for identifying rib numbers among hundreds of CT slices to designate abnormal region, whereas annotated slice enables radiologists to skip superfluous procedure

Precise and instant identification of the chest abnormalities is crucial for establishing a treatment plan and monitoring progression of the heart, lungs, major blood vessels, and bone structures among the hundreds of chest Computed Tomography (CT) slices [1–5]. The ribs are valuable reference points for interpreting chest CT, as they encircle the thorax in a vertical alignment [6, 7]. The anatomical characteristics of ribs enable radiologist to directly pinpoint abnormalities by utilizing the rib number among substantial CT slices [8, 9]. Computer-aided rib numbering liberates radiologists from repeatedly examining non-annotated CT slices as shown in Fig. 1a, by representing rib annotations on every substantial CT slice Fig. 1b. The rib annotations enable radiologists to localize the affected areas swiftly, however, annotation accuracy highly depends on preceding rib segmentation performance in time-constrained clinical settings.

Rib segmentation on chest CT is a challenging task due to the complex chest anatomy which comprises numerous bone structures (e.g., scapulae, spine, ribs, clavicles) and anatomical variability across patients, obscuring clear rib boundaries. Recent advances in Deep Learning (DL) shed light on precise rib segmentation by learning diverse rib features across anatomical variations [10–19], however, the representation capability of DL is restrained to partial rib context (e.g., 2D slice, 3D patch-wise). The coarse-level rib segmentation is indispensable, attributed to the high computational cost for processing entire chest CT volume. This limitation leads to incomplete 3D rib construction and oversegmentation of similar or neighboring bone structures. The rib annotations from coarse ribs strive to provide the robust and reliable reference points for chest CT interpretation in clinical practice.

DL-driven chest CT rib segmentation is broadly categorized into patch or slice-wise [10, 12, 14, 15], and point-wise segmentation [11, 13]. Patch and slice-wise segmentation focus on learning rib anatomy from localized image regions. The first DL method utilizes an ensemble of Faster R-CNN and 3D U-Net, aggregating the partial predictions from 2D axial slices and 3D patches of CT volume to reconstruct ribs [10]. Ensemble method examines local rib context, while inducing high computational costs to obtain and merge numerous partial predictions. Meanwhile, slice-wise segmentation effectively learns the anatomical structures of ribs on the 2D axial slice, reducing computational cost [12, 14]. This slice-wise method struggles to detect the excessively short or floating ribs (i.e., the 11th and 12th ribs). Multi-axial segmentation investigates diverse aspects of ribs from three different anatomical planes (i.e., axial, coronal, sagittal planes) [15]. This method significantly increases computational costs due to threefold slice-wise inference for a single CT volume, resulting in inefficient large-scale or real-time applications. The patch or slice-wise segmentation strives to learn overall anatomical structure of ribs by combining hundreds of segmentation pieces. This strategy often leads to oversegmentation (e.g., transverse process), rib omission (e.g., misaligned or floating ribs) and a long inference time, restricting the clinical practicality.

Point-wise segmentation provides an alternative strategy to effectively learn the entire rib anatomy by computing point-cloud of 3D coordinates from CT volume. Point-cloud downsampling is adopted to decrease computational costs by processing sparse but informative point-cloud which captures the global rib structure [11]. This sparse prediction leads to omission of short ribs and requires complex post-processing for fine-grained segmentation results, yielding inconsistent prediction results. To mitigate post-processing dependency, point-batch processing divides dense point-cloud into equal size coarse batches and consolidates batch-wise model predictions [13]. This method improves the output stability, however, the DL model itself still suffers from learning local details of ribs due to coarse point-batch level representation. The point-wise segmentation faces challenges in learning capability to acquire the fine structural and boundary features of ribs. Previous studies commonly face challenges in capturing local and global anatomical characteristic of ribs simultaneously. Slice and patch-wise DL segmentation focus on local ribs, whereas point-wise segmentation only aims to learn global ribs. To this end, a novel rib segmentation method is necessary for capturing both local and global anatomical characteristics of ribs.

**Fig. 2:**
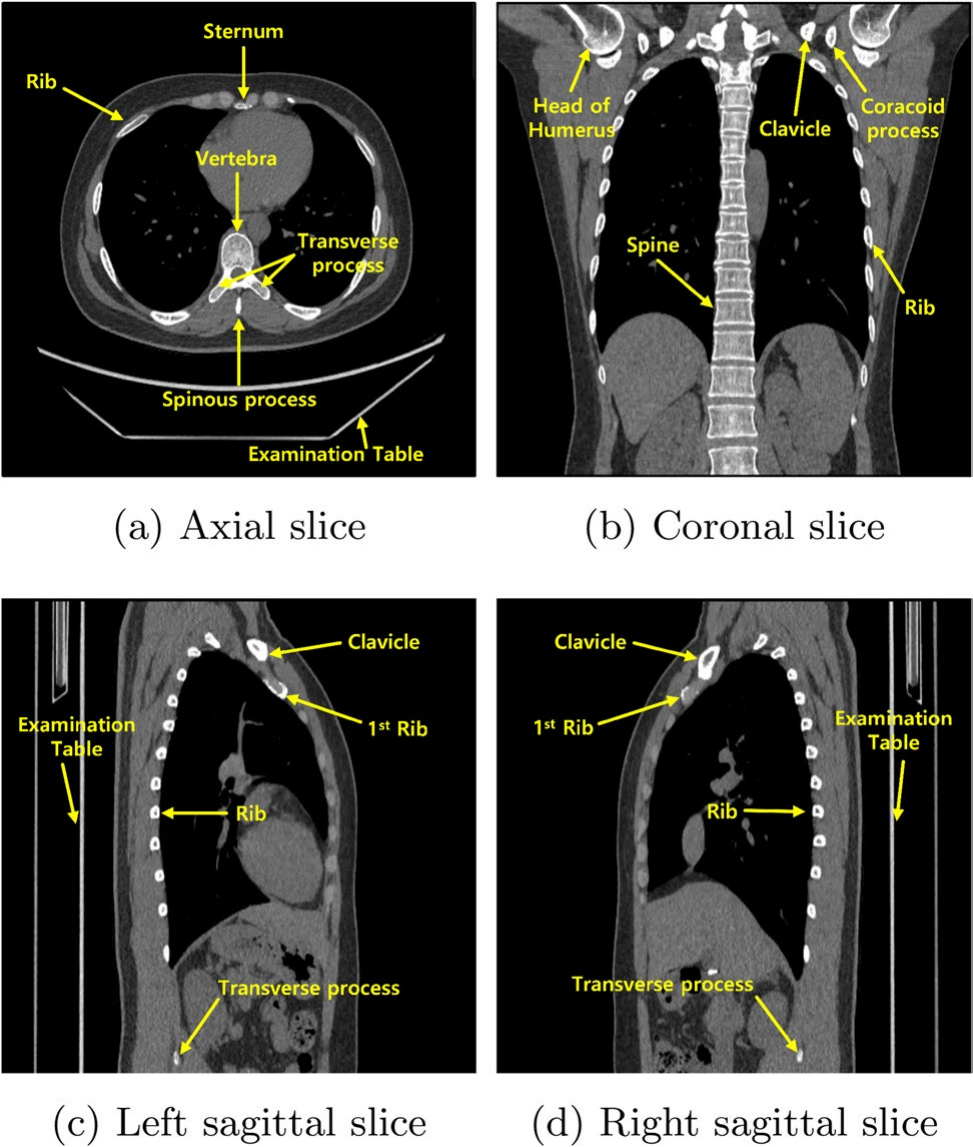
Representative examples of multiplanar slices of chest CT. While axial and coronal views contain overlapping structures that hinder rib isolation, the sagittal slices clearly delineate all twelve rib pairs with minimal anatomical interference, offering an optimal perspective for rib localization and segmentation

To solely utilize the advantages of DL, this study exploits the anatomical structure of 12 rib pairs. The ribs are vertically aligned and encircle the thorax in a curved shape [20], providing optimal visibility in the sagittal slice for their localization, as shown in Fig. 2. The axial Fig. 2a and coronal slice Fig. 2b require the DL model to explore various CT slices for identifying every concealed rib, whereas the left Fig. 2c and right sagittal slices Fig. 2d near the spine reveal all rib pairs simultaneously. Rib segmentation on sagittal slices enables DL model to comprehensively capture the entire spatial arrangement of all ribs with the least number of CT slices, while robustly accounting for diverse anatomical variations. Leveraging the anatomical guidance of 2D sagittal ribs, we propose Dual sagittal-guided Coarse-to-fine Rib Segmentation (DCRS) for 3D rib segmentation in chest CT. The proposed DCRS initially identifies two sagittal slices adjacent to the left and right spine boundaries. The coarse rib segmentation stage yields reliable left and right rib predictions that embed global rib context within local chest CT regions. The fine rib construction stage selects seeds from each sagittal rib prediction and explores connected regions of seeds in a recursive manner. This coarse-to-fine segmentation approach produces assured 3D rib segmentation results for robust sequential numbering. The major contributions of this study are summarized as follows:

- To the best of our knowledge, this study is the first coarse-to-fine 3D rib segmentation framework for assured rib numbering. The proposed DCRS streamlines 3D rib segmentation task by leveraging DL-based 2D coarse rib segmentation followed by fine 3D rib construction.
- The proposed Region of Non-Interest (RONI) removal suppresses soft tissues and isolates individual ribs from the thorax, providing stable anatomical boundaries for robust pivot slice selection in the 2D coarse rib segmentation.
- The proposed Six-Neighborhood Outward Flood-Filling (SNOFF) expands 2D sagittal rib seeds within the rib-isolated volume, ensuring voxel-wise connectivity while reducing non-rib predictions.
- Comprehensive performance evaluation of the proposed DCRS against previous state-of-the-art methods are carried out, including improvements of Dice 3.1%, Intersection over Union 4.1%, accuracy 2.1%, precision 3.4%, recall 3.9%, and specificity 2.1%.
- Sequential rib numbering embeds voxel-wise rib number and laterality annotations on chest CT slices. It takes only 8.9 seconds from DCRS to sequential numbering on average, showcasing 44% improvement compared to the previous state-of-the-art method.

**Fig. 3:**
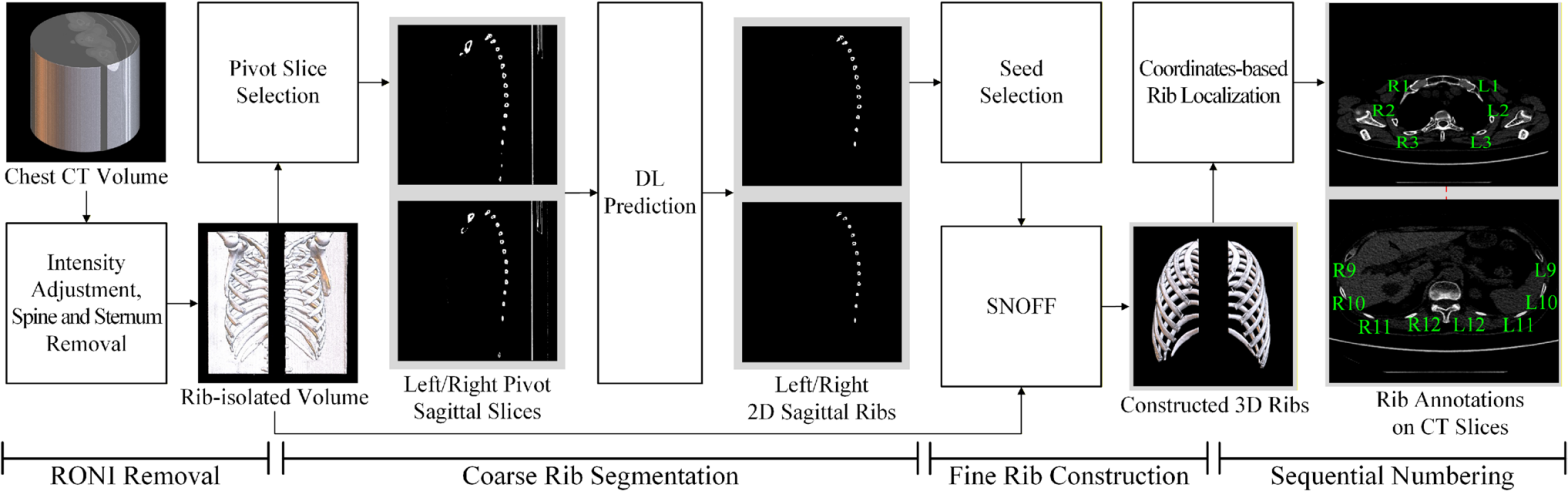
Overall architecture of the proposed DCRS and sequential numbering

**Fig. 4:**
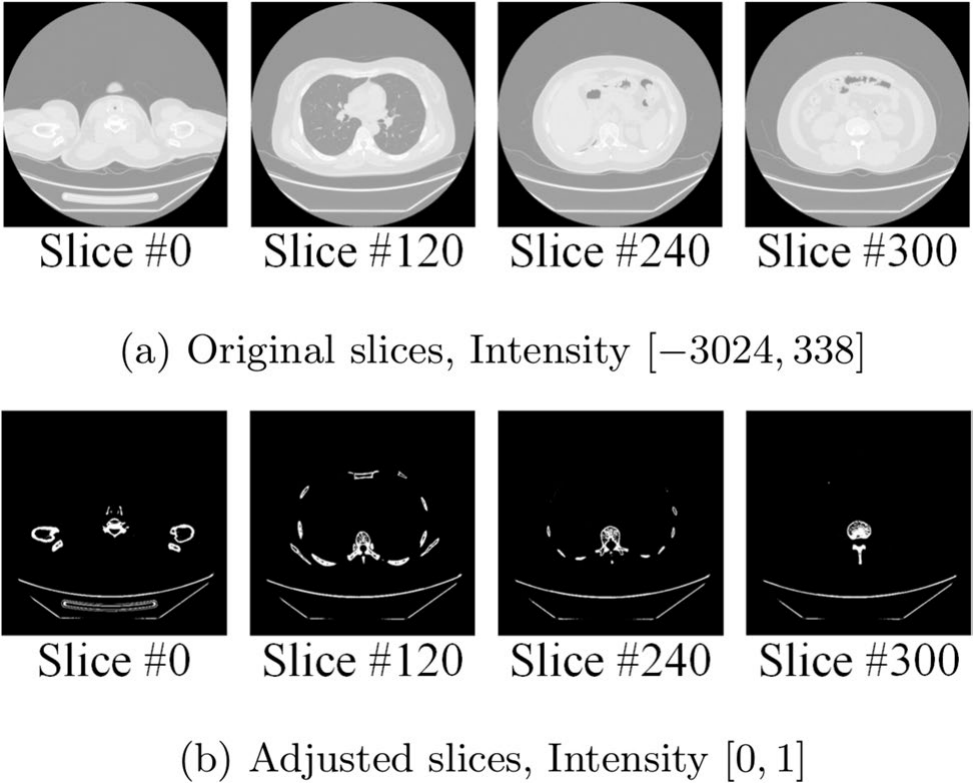
Intensity adjustment of chest CT slices. Adjusted slices show enhanced contrast for bone structures and effective removal of soft tissue components

## Methods

The proposed Dual sagittal-guided Coarse-to-fine Rib Segmentation (DCRS) and sequential numbering comprises four stages, as illustrated in Fig. 3: preprocessing, coarse rib segmentation, fine rib construction, and sequential numbering. The preprocessing stage contains two steps of intensity adjustment and spine removal, eliminating Regions Of Non-Interest (RONI) from the chest CT volume. The coarse rib segmentation stage takes two pivot slices from the removed boundaries of rib-isolated volume and yields sagittal rib predictions. The fine rib construction stage selects seed voxels from each sagittal rib and constructs 3D ribs with the proposed Six-Neighborhood Outward Flood-Filling (SNOFF) algorithm. Finally, the sequential numbering stage embeds voxel-wise rib annotations within chest CT volume, based on 3D coordinates of each rib.

**Fig. 5:**
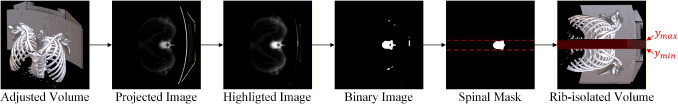
Intermediate outputs of RONI removal stage. The red lines in spinal mask denote min and max *y* coordinates of the personalized kernel, which is described with red box in rib-isolated volume

### Region of non-interest removal

The chest CT volume exhibits a broad and variable pixel intensity range, resulting in clearer visibility of soft tissues compared to bones in CT slices, as shown in Fig. 4a. Moreover, this extensive intensity range increases computational costs during the subsequent segmentation stage. It is essential to adjust the intensity of CT volumes for enhancing rib visibility and reducing unnecessary computational complexity. The CT intensities are manifested in Hounsfield Units (HU), reflecting the radiodensity of different body tissues [21]. The CT windowing technique adjusts the HU range to highlight specific anatomical structures for easier identification and analysis [22]. This process utilizes two parameters, Window Width (WW) and Window Level (WL). WW defines the range of HU values displayed, while WL establishes the median of this range. In this study, WW and WL are set as 50 and 175, respectively.

The resultant voxel intensity range is confined to the interval between the minimum and maximum window values, clipping the out-of-range values to the nearest boundary. The intensity range is further normalized to [0,1] for training stability and fast convergence of the DL model [23, 24]. The chest CT slices after windowing and normalization are depicted in Fig. 4b. The intensity adjustment significantly enhances visibility of bone structures, while minimizing the representation of other body tissues.

The intensity adjustment step effectively reduces the CT data complexity. The adjusted volume only contains bone structures and examination table, however, the direct connection among spine, sternum, and ribs disturbs assured 3D rib construction. The separation of interconnected bones is necessary not only to isolate individual ribs for 3D construction but also to provide clear boundaries for pivot selection during 2D segmentation. The removal process for each chest CT consists of 5 steps, as shown in Fig. 5.

The first step aggregates voxel intensities (*x*, *y*, *z*) along the *z*-axis of a CT volume *V*, yielding a projected image $P(x,y)=\sum _{z} V(x,y,z)$, which corresponds to summing the intensities of all axial slices along the vertical axis. This projection enhances the visibility of structures like the spine, which span multiple slices and thus accumulate higher intensity values. The pixel intensity distribution in spinal area is broad due to the distinct position of individual vertebrae and anatomical differences. In the second step, spine highlighting replaces each pixel intensity in the projected image with the median of its neighbor pixels, resulting in a highlighted image $H(x,y) = \textrm{median} \left\{ P(x+i,y+i) \,|\, -k \le i \le k \right\}$ where *k* is the neighborhood radius. This process smooths the intensity distribution and enhances the visibility of the spine, making it more consistently distinguishable from other anatomical structures.

An adaptive threshold *t* converts the highlighted image *H* into binary image $B(x,y)=1\ \text {if }H(x,y)\ge t,\;0\text { otherwise}$. The binary image *B* contains noises which have similar vertical length with spine, necessitating noise removal by morphological opening $O(x,y) = \delta (\epsilon (D(x,y)))$ followed by closing $C(x,y) = \epsilon (\delta (O(x,y))$, where $\epsilon$ and $\delta$ denote erosion and dilation, respectively. In the fourth step, these operations help remove small noisy components and fill minor gaps in the spinal area, resulting in a cleaner and more accurate binary representation of the spine.

In the final step, the refined spinal mask is used to define a personalized 3D kernel for spine and sternum removal. The minimum and maximum *y* coordinates in the segregated spinal area *C* are utilized to create a personalized 3D kernel. The kernel has a shape of $(512, y_{max}-y_{min},N)$, where *N* denotes the axial slice number of each CT volume. This kernel has the same width and depth as the CT volume but is confined vertically to the spinal region. It is extended across all slices and used to zero out the corresponding voxel values, thereby eliminating both the spine and the sternum and truncating individual ribs. The resultant rib-isolated volume enables robust pivot identification and anatomically consistent rib segmentation in subsequent stages.

### Coarse rib segmentation

**Fig. 6:**
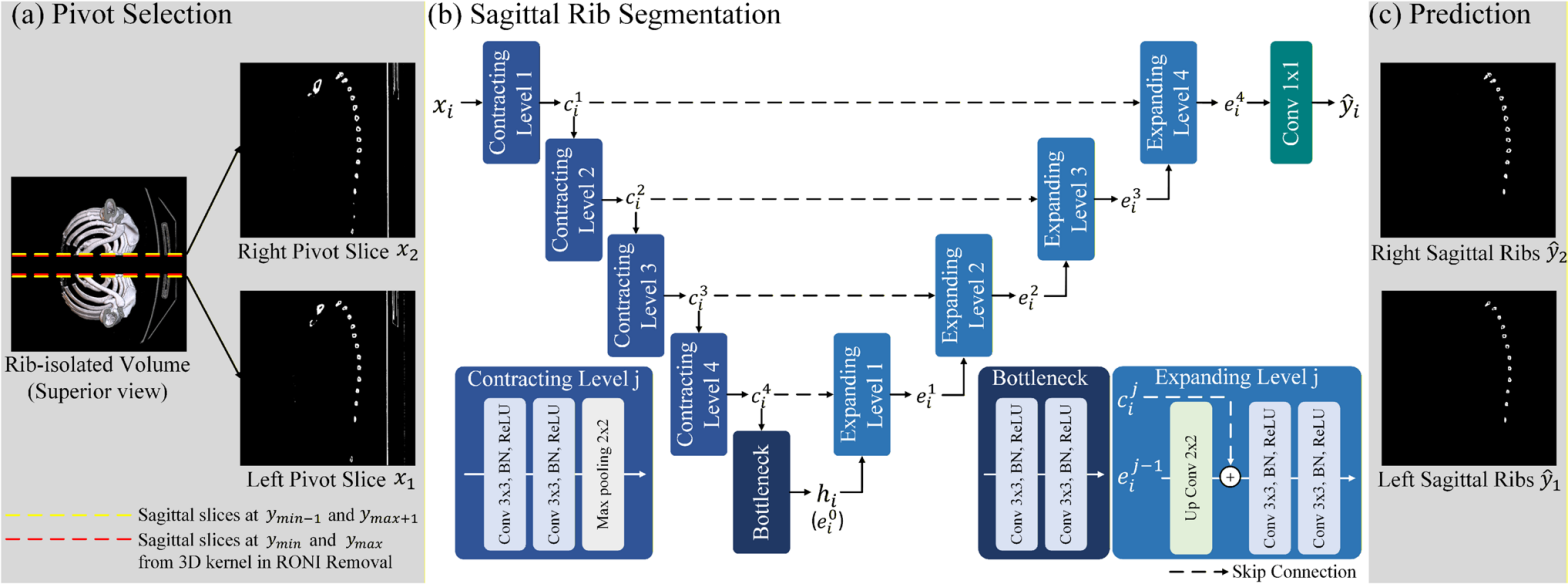
Rib segmentation on selected left and right sagittal slices through the contracting and expanding operations of U-Net. (**a**) describes pivot selection utilizing the boundaries of removed region (red lines). (**b**) represents the architecture of U-Net which intakes two sagittal slices $x_1$ and $x_2$. (**c**) illustrates the left and right sagittal rib predictions $\hat{y}_1$ and $\hat{y}_2$

Let the personalized 3D kernel define the removed spine region boundaries along the sagittal axis, which are denoted as sagittal slices at index $y_{min}$ and $y_{max}$ in Fig. 5. We select two sagittal pivot slices as the immediate neighbors of the removed region. The left pivot $x_1$ is the sagittal slice at index $y_{min-1}$ and the right pivot $x_2$ is at index $y_{max+1}$. The U-Net model [25] intakes left and right pivots, propagating them through contracting and expanding paths, as shown in Fig. 6. The contracting path $C(\cdot )$ transforms the input slice $x_i$ into a high-dimensional feature representation $h_i$ as the network depth increases:

1 $$\begin{aligned} h_i = C(x_i), \quad i \in \{1, 2\}, \end{aligned}$$

where $C(\cdot )$ consists of four $\text {ContractingLevel}_j$ and a bottleneck layer. Each $\text {ContractingLevel}_j$ comprises two sequences of (Conv $3 \times 3$, BN, ReLU) followed by a $2 \times 2$ max pooling operation, yielding contracting feature $c_i^j$ at level *j*. Bottleneck intakes $c_i^4$ and produces $h_i$ through duplicated sequences of (Conv $3 \times 3$, BN, ReLU). The expanding path $E(\cdot )$ restores the feature representation $h_i$ to sagittal rib predictions $\hat{y}_i=E(h_i)$, where $E(\cdot )$ comprises four $\text {ExpandingLevel}_j$ and following Conv $1 \times 1$. The initial input for expansion is set as $e_i^0=h_i$, and each $\text {ExpandingLevel}_j$ receives $e_i^{j-1}$ and $c_i^j$ to refine the spatial representation of ribs $e_i^j$:

2 $$\begin{aligned} e_i^j = \text {ExpandingLevel}_j \left( e_i^{j-1}, c_i^j \right) , \end{aligned}$$

where $\text {ExpandingLevel}_j$ consists of Up-Conv $2 \times 2$ and following two sequences of (Conv $3 \times 3$, BN, ReLU). The final layer takes $e_i^4$ to predict the left and right sagittal ribs $\hat{y}_1$ and $\hat{y}_2$:

3 $$\begin{aligned} \hat{y}_i = \sigma (\text {Conv}{1 \times 1}(e_i^4)), \end{aligned}$$

where $\sigma$ is the sigmoid function. The $\hat{y}_1$ and $\hat{y}_2$ are predicted by a DL model which learns comprehensive global spatial as well as local structural information of ribs. The model identifies every coarse 2D rib with only two sagittal slices. This lightweight rib identification approach provides robust and precise cornerstones for 3D rib construction.

The sagittal rib segmentation task aims to produce morphologically similar segmentation results with labels and classify all pixels into ribs or non-ribs. We employ a combined loss function of Binary Cross-Entropy (BCE) and Intersection over Union (IoU) loss to train the U-Net effectively [26, 27]. The BCE loss penalizes pixel-wise classification errors:

4 $$\begin{aligned} \mathcal {L}_{\text {BCE}} = -\frac{1}{N} \sum _{i=1}^{N} \left[ y_i \log (\hat{y}_i) + (1 - y_i) \log (1 - \hat{y}_i) \right] , \end{aligned}$$

where $y_i \in \{0,1\}$ is the ground truth label, $\hat{y}_i \in [0,1]$ is the predicted probability, and *N* is the number of pixels. The IoU loss complements the BCE loss by optimizing the overlap between the segmentation mask and the ground truth label:

5 $$\begin{aligned} \mathcal {L}_{\text {IoU}} = 1 - \frac{\sum _{i=1}^{N} y_i \hat{y}_i}{\sum _{i=1}^{N} \left( y_i + \hat{y}_i - y_i \hat{y}_i \right) + \varepsilon }, \end{aligned}$$

where $\varepsilon$ is a small constant added for numerical stability. The final segmentation loss $\mathcal {L}_{\text {seg}}$ is defined as a weighted sum of BCE and IoU losses:

6 $$\begin{aligned} \mathcal {L}_{\text {seg}} = \alpha \mathcal {L}_{\text {BCE}} + \beta \mathcal {L}_{\text {IoU}} \end{aligned}$$

In this study, both $\alpha$ and $\beta$ are set to 0.5 to give equal importance to pixel-wise accuracy and morphological similarity during model training.

### Fine rib construction

The coarse left and right sagittal rib predictions provide seed voxels for the proposed Six-Neighborhood Outward Flood-Filling (SNOFF) algorithm to construct fine-grained 3D rib volume. SNOFF iteratively expands a set of predefined seed voxels *s*, gradually filling a zero-padded volume *R*, as described in Algorithm 1. The number of neighborhood voxels is set to six, as six is the minimum number of voxels to cover *x*, *y*, and *z* direction without computational redundancy. The rib-isolated volume is converted to binary volume, enabling SNOFF to operate on dense voxel-level connectivity.

**Algorithm 1:**
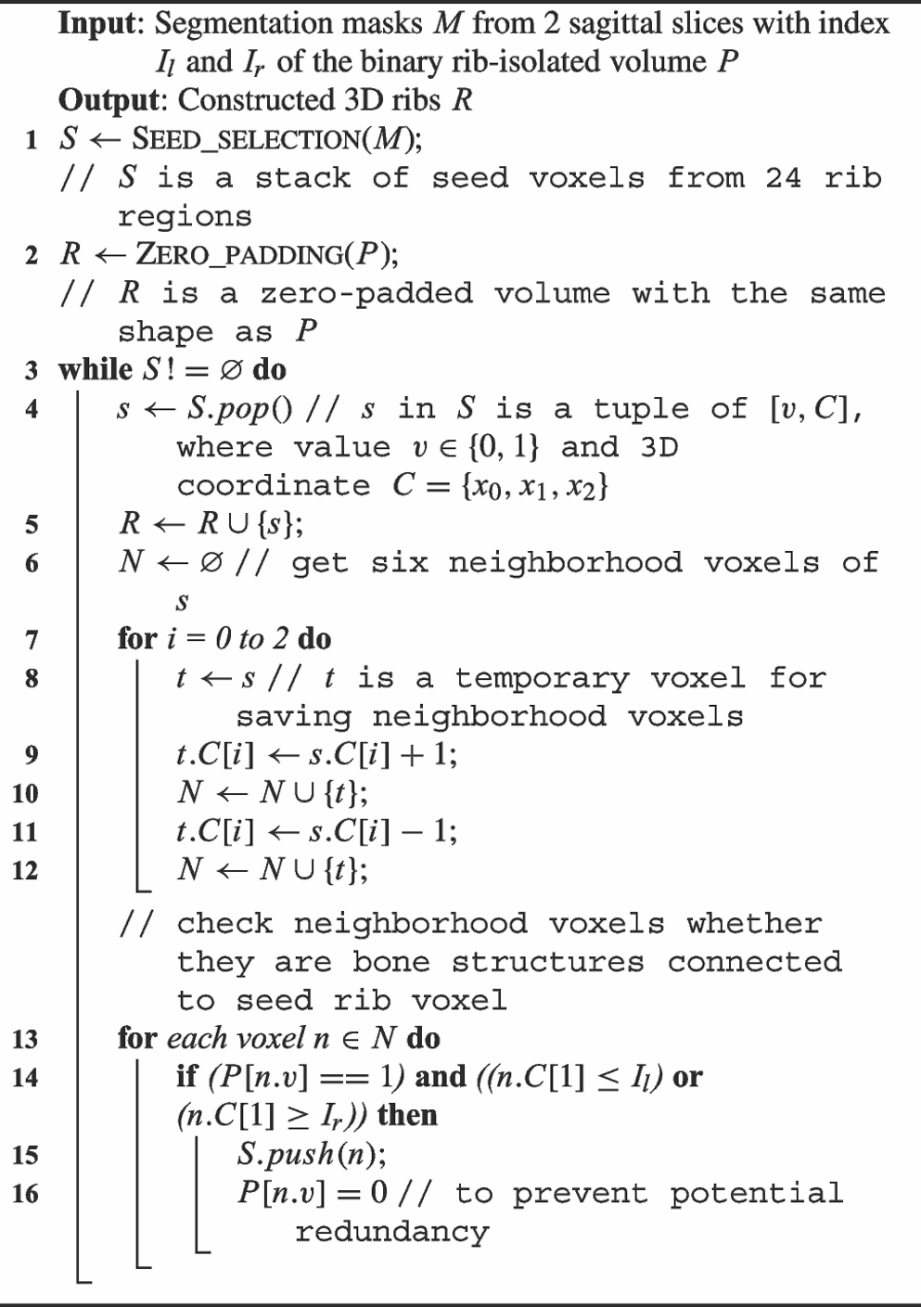
Six-Neighborhood Outward Flood-Filling (SNOFF)

Seed Selection automatically selects a representative pixel from each sagittal rib. The 2D pixel coordinates of seeds are then converted into 3D voxel coordinates. The 2D coordinates (*x*, *y*) correspond to voxel coordinates (*y*, *z*), and the sagittal slice number serves as the voxel coordinate *x*. The resultant seed voxels *s* are initially added to *R*. SNOFF identifies additional rib voxels by examining the six adjacent voxels *n* surrounding each seed voxel *s* along the *x*,*y* and *z* directions. If a neighboring voxel *n* has a value of 1 in the rib-isolated volume *P*, it is classified as a rib voxel and added to *R*. The rib voxel takes a role of next seed *s*. SNOFF recursively explores these newly identified rib voxels outward until no further connected voxels remain to be classified. The resultant fine-grained 3D ribs benefit from the robust sagittal rib segmentation and rib isolation during the preprocessing stage.

**Fig. 7:**
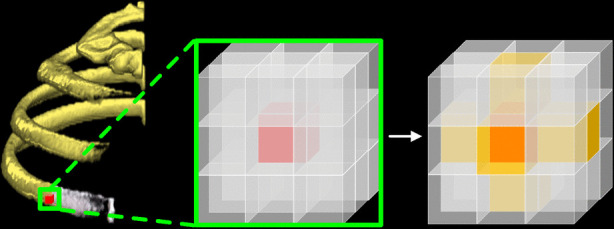
Seed expansion for constructing 3D ribs through the SNOFF. The yellow and white regions in the right 4th rib indicate voxels already classified as a rib and those awaiting flood-filling, respectively. The green box magnifies the seed (red voxel) and its 26 adjacent voxels. Yellow voxels are six neighborhoods of the seed

The proposed SNOFF algorithm provides a simple yet effective strategy for constructing fine-grained 3D rib volumes from coarse 2D predictions on two sagittal slices, as illustrated in Fig. 7. SNOFF leverages anatomically guided sagittal rib segmentation result to extract seed voxels and iteratively expand them in 3D space. The expansion is confined to six neighboring voxels and is performed within a rib-isolated volume from which non-rib regions such as soft tissues and the spine have been removed. This allows SNOFF to grow only within rib candidate areas while minimizing leakage into irrelevant structures. The combination of anatomically-informed seeding and constrained 3D neighborhood expansion yields accurate and noise-resistant 3D ribs with low computational cost in clinical CT workflows.

### Sequential rib numbering

Sequential numbering utilizes the 3D coordinates (*x*, *y*, *z*) of the constructed rib volume. It is crucial to separate all ribs into left and right sides for precise numbering. The maximum *x* coordinates of all ribs are arranged in ascending order, as maximum coordinate is robust to the spinal deformity or patient position. The largest distance among consecutive *x* coordinates serves as the dividing point. Ribs are classified into left and right ribs depending on the dividing point. This laterality is embedded in all voxels of the individual rib. The ribs on each side are sorted again in descending order according to their maximum *z* coordinate. Individual sorted ribs are sequentially numbered from 1. All the voxels depicting ribs become informative with rib number and its laterality. This voxel-level dense embedding enables every coronal, sagittal, and axial chest CT slice to represent the precise positions of all ribs.

## Results

### Datasets and experimental setting

This study utilizes 460 pairs of chest CT volumes and rib segmentation labels in the the RibFrac dataset [28] and RibSeg dataset [11], respectively. The RibFrac dataset was collected at the Fudan University Huadong Hospital over a 2-year period. Two CT scanners (GE Healthcare Revolution CT, Siemens Healthcare Somatom Definition Flash) performed chest-abdomen CT with the following parameters: 120 kVp, 100-200 mAs, pitch 0.75-1.5, collimation 11.25 mm, and and slice thickness 1-1.25mm. Additional information about the data is detailed in [11, 28], such as gender ratio and age. The RibSeg dataset contains binary rib segmentation labels for each corresponding CT volumes in RibFrac dataset. The segmentation labels are extracted with semi-automatic pipeline, comprising morphological algorithms and manual refinement. Each data pair contains *N* axial slices with a resolution of $[512\times 512]$ pixels, where *N* typically ranges from 300 to 400.

Our experiments divide 460 pairs of chest CT volumes and rib segmentation labels into three subsets. The U-Net model training and validation utilize 410 pairs and 3D rib segmentation performance is tested on 50 pairs. The training and validation set undergo sagittal slice extraction by identifying the slices which represent the 12 ribs on the label. The resultant 820 slice pairs are resized to $[512\times 512]$ resolution and divided into training and validation sets with an 80:20 ratio, to monitor the model performances in unseen cases while learning diverse rib features [29, 30]. The U-Net model is trained with a learning rate of 0.001, a batch size of 12, and 200 epochs. Adam optimizer supports balanced minimization for BCE and IoU losses [31]. An early stopping algorithm prevents the model overfitting to training set, terminating the training process when the validation loss increases for five consecutive epochs [32]. Our proposed method is implemented with PyTorch and CUDA libraries on a hardware environment comprising an Intel i9 CPU, 32GB RAM, and RTX 3090Ti GPU.

Performance evaluation is divided into 3D rib segmentation and sequential numbering. Rib segmentation performance is measured with morphological similarities (Dice, IoU) and classification metrics (Accuracy, Precision, Recall, Specificity) by comparing 3D constructed ribs with labels [17, 19]. The six metrics are defined as:

7 $$\begin{aligned} \text {Dice} = \frac{2TP}{2TP + FP + FN} \end{aligned}$$

8 $$\begin{aligned} \text {IoU} = \frac{TP}{TP + FP + FN} \end{aligned}$$

9 $$\begin{aligned} \text {Accuracy} = \frac{TP + TN}{TP + TN + FP + FN} \end{aligned}$$

10 $$\begin{aligned} \text {Precision} = \frac{TP}{TP + FP} \end{aligned}$$

11 $$\begin{aligned} \text {Recall} = \frac{TP}{TP + FN} \end{aligned}$$

12 $$\begin{aligned} \text {Specificity} = \frac{TN}{TN + FP} \end{aligned}$$

where *TP*, *TN*, *FP*, and *FN* denote True Positive, True Negative, False Positive, and False Negative voxels, respectively. We also calculate the FLoating point Operations Per Second (FLOPS) and the number of model parameters to evaluate computational efficiency [18].

**Table 1:** Performance comparison of different rib segmentation methods with 50 testset on RibSeg dataset

**Methods**		**Dice**	**IoU**	**Accuracy**	**Precision**	**Recall**	**Specificity**
Coarse	Wu et al. [10]	81.58±6.07	72.92±6.92	92.87±0.36	82.35±6.12	86.51±8.09	93.34±0.41
		(79.90-83.26)	(71.00-74.84)	(92.77-92.97)	(80.65-84.05)	(84.27-88.75)	(93.23-93.45)
	Seol et al. [12]	87.42±3.11	84.76±3.44	96.94±0.22	88.51±4.57	89.34±7.04	97.02±0.25
		(86.56-88.28)	(83.81-85.71)	(96.88-97.00)	(87.24-89.78)	(87.40-91.28)	(96.96-97.08)
	Jin et al. [13]	89.17±5.21	82.56±5.76	97.26±0.19	87.44±5.41	90.85±7.62	97.52±0.23
		(87.73-90.61)	(80.97-84.15)	(97.21-97.31)	(85.94-88.94)	(88.75-92.95)	(97.46-97.58)
	Leonov et al. [14]	85.24±3.86	79.27±4.32	95.82±0.27	86.98±4.86	84.67±7.13	96.39±0.31
		(84.17-86.31)	(78.07-80.47)	(95.75-95.89)	(85.64-88.32)	(82.75-86.59)	(96.31-96.47)
	Kim et al. [15]	83.84±2.94	90.03±2.58	97.85±0.21	89.37±3.72	91.21±6.41	97.91±0.25
		(89.32-90.74)	(83.02-84.66)	(97.79-97.91)	(88.34-90.40)	(89.44-92.98)	(97.86-97.96)
Coarse-to-fine	**Proposed DCRS**	**92.83**±**2.40**	**88.21**±**3.21**	**99.93**±**0.06**	**92.43±4.07**	**94.73**±**7.12**	**99.96**±**0.06**
		(92.16-93.49)	(87.32-89.10)	(99.91-99.95)	(91.28-93.58)	(92.71-96.75)	(99.94-99.98)

Values are shown as mean±SD with 95% CI on the line below. The best and second best results are highlighted in **bold** and underline, respectively. Paired *t*-test confirmed that the proposed DCRS outperformed the second best method [15] by at least 2.1% across six evaluation metrics with all *p*-values$< 0.001$

The rib numbering accuracy $\text {Acc}_{\text {num}}$ evaluates whether the predicted rib number and laterality $\widehat{\Gamma }_{v,n}$ exactly match the ground truth ${\Gamma }_{v,n}$ across *N* axial slices of each CT volume *V*. A volume is considered correct only if all slice-level predictions are perfectly accurate. The metric is defined as:

13 $$\begin{aligned} \text {Acc}_{\text {num}}&= \frac{1}{V} \sum _{v=1}^{V} \textbf{1}\!\Bigl (\, \forall \,n \in \{1,\dots ,N\}:\; \widehat{\Gamma }_{v,n} = \Gamma _{v,n} \Bigr ), \end{aligned}$$

where $\textbf{1}(\cdot )$ is the indicator function that returns 1 if the condition holds true, and 0 otherwise. Finally, we calculated the average throughput from rib segmentation to sequential numbering across the testset.

### Experimental result

We compare the proposed DCRS against the previous state-of-the-art 3D rib segmentation methods, as shown in Table 1. The DCRS shows Dice 92.83% and IoU 88.21%, outperforming the second best methods [12, 15] by 3.1% and 4.1%, respectively. The DCRS assures voxel-wise rib connectivity with fine-graining 3D construction by the SNOFF algorithm, yielding the best segmentation performance. The other methods do not check the voxel-wise connectivity during segmentation, as they focus on processing 2D slices, 3D patches, and point-cloud.

Voxel-level classification metrics are further measured for in-depth analysis. The proposed DCRS also surpasses all other methods across the four voxel-level classification metrics. Our method exceeds the second best method [15] by 2.1% on both accuracy and specificity. The sagittal rib segmentation ensures that the DL model provides robust rib seeds for the following SNOFF algorithm, resulting in precise classification of rib and background voxels. The DCRS also improves 3.4% precision and 3.9% recall compared to the second best method [15], respectively. The RONI removal in the preprocessing stage eliminates posterior ribs, improving overall accuracy and specificity while minimally impacting precision and recall. Paired *t*-test confirmed that the proposed DCRS outperformed the second best method [15] by at least 2.1% across six evaluation metrics with all *p*-values $< 0.001$.

**Fig. 8:**
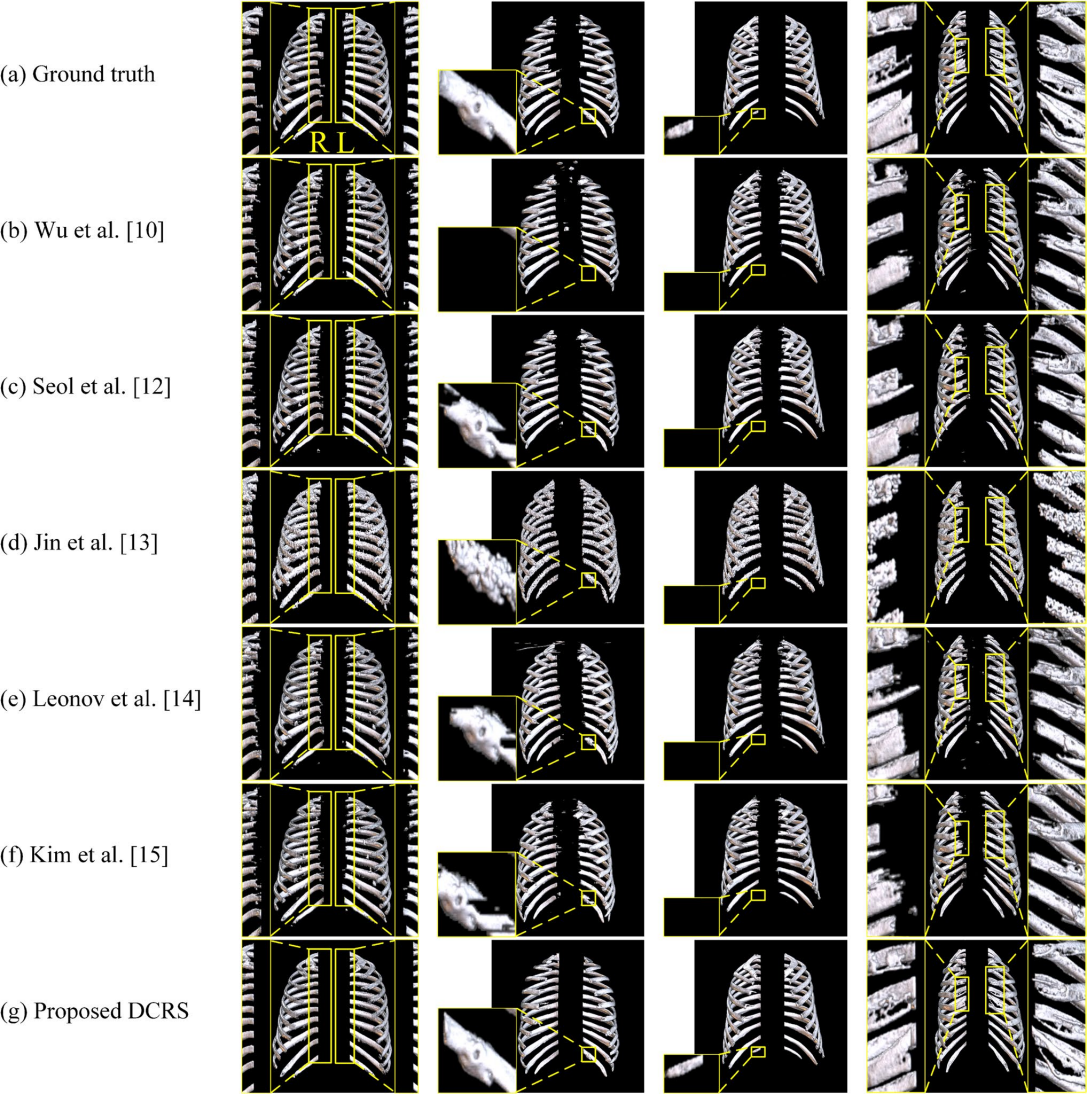
Comparison of segmentation results for four challenging rib cases: scoliosis, rib fractures, excessively short floating ribs, and costal cartilages. Previous methods Fig. 8(**b**), 8(**c**), 8(**d**), 8(**e**), and 8(**f**) exhibit over-segmentation, missing ribs, or inclusion of noise. In particular, they fail to detect fractures or short floating ribs and struggle with costal cartilages. In contrast, the proposed DCRS Fig. 8(**g**) provides anatomically accurate and complete segmentation across all cases

Segmentation results of the DCRS and the other rib segmentation methods are illustrated in Fig. 8 with the corresponding ground truth labels. This visualization demonstrates the proposed DCRS predicts the most accurate rib segmentation mask among all methods. There are four different cases, as shown in Fig. 8(a). The first column shows a case of scoliosis in the original CT volume. The transverse process and costal cartilages are shown in the outputs of Fig. 8(b) and 8(c). The right 3rd and left 5th ribs are shortly reconstructed in Fig. 8(d). Noises are contained in Fig. 8(e) and Fig. 8(f). The proposed DCRS Fig. 8(g) misses some rib voxels near the spinal region, however, DCRS output does not contain the noise.

The second case involves healed fractures of the left floating ribs. The 12th rib pairs are invisible and the fragments of vertebrae and mandible are contained in the mask of Fig. 8(b). The output of Fig. 8(c) represents the left 12th rib broken in two and the segmentation failure of right 12th rib. Also, the left 12th rib construction is incomplete in Fig. 8(d). The outputs of Fig. 8(e) and 8(f) represent every rib pair, however, the 12th rib pair shows partial omission of rib voxels. Furthermore, the noise inclusion potentially affects the subsequent sequential numbering. The DCRS Fig. 8(g) is robust to fracture without noise and segmentation failure.

The third case contains excessively short floating ribs. DL models can misidentify short floating ribs as transverse processes because they are not connected to the sternum. All methods Fig. 8(b). Figure 8(c), 8(d), 8(e), and 8(f) fail to segment the right 12th rib, and also the left 12th rib. Moreover, the left 2nd and 3rd anterior ribs are omitted in Fig. 8(d). The proposed DCRS Fig. 8(g) successfully recovers all floating ribs with high fidelity, leveraging coarse-level sagittal-guidance and fine-level rib construction with SNOFF algorithm.

**Table 2:** Performance comparison of different rib numbering methods with 50 testset on RibSeg dataset

Methods	Accuracy (Success case)	Throughput	#Params	GFLOPs
Wu et al. [10]	86% (43/50)	3.42	75.20M	155.1
Seol et al. [12]	94% (47/50)	4.05	1.939M	**13.67**
Jin et al. [13]	96% (48/50)	4.67	**1.891M**	21.81
Leonov et al. [14]	94% (47/50)	3.88	3.250M	15.06
Kim et al. [15]	98% (49/50)	1.32	93.14M	175.9
Proposed DCRS	**100% (50/50)**	**6.74**	1.939M	**13.67**

Throughput is defined as the number of processed CT volumes per minute. The best and second best results are highlighted in **bold** and underline, respectively

The final case is the costal cartilage calcification. The outputs of Fig. 8(b), 8(c), 8(e), and 8(f) fail to exclude the costal cartilage, struggle to construct left 1st and 2nd ribs successfully, and even contain transverse process with vertebral fragments. The only method Fig. 8(d) is robust in this case, but omits the left 1st rib. The DCRS Fig. 8(g) includes costal cartilage as well, however, DCRS successfully constructs 12 ribs without noise. The previous state-of-the-art methods [10, 12–15] focus solely on the local or global structure of ribs, without fine-graining stage which complements their coarse slice, patch, point-wise predictions. The limited learning of entire rib anatomy leads to confusion of ribs with similar anatomical structures and rib omission. In contrast, the proposed DCRS yields reliable 3D rib segmentation result by focusing on the global anatomical features of every rib pair within local regions of chest CT. The DCRS maximizes the advantage of DL which captures diverse rib features and enables the subsequent SNOFF to construct robust 3D ribs without noises, benefitting from the coarse-to-fine segmentation approach.

The sequential rib numbering performance highly depends on the 3D rib segmentation results, as shown in Table 2. Sequential numbering utilizing DCRS output achieves a perfect match with the label, surpassing the second best method [15] by 2%. The failure cases of [10, 12–15] are due to missing ribs and oversegmented structures, as shown in Fig. 8. DCRS and sequential labeling yields 6.74 annotated CT volumes per minute, showcasing 44% improvement compared to the second best method [13]. The proposed DCRS leverages only two sagittal slices for identifying 12 rib pairs with lightweight U-Net compared to previous patch, slice, and point-wise segmentation methods. This design choice drastically reduces both computational burden and data redundancy, achieving second lowest parameter count and the lowest GFLOPs among all compared methods.

**Fig. 9:**
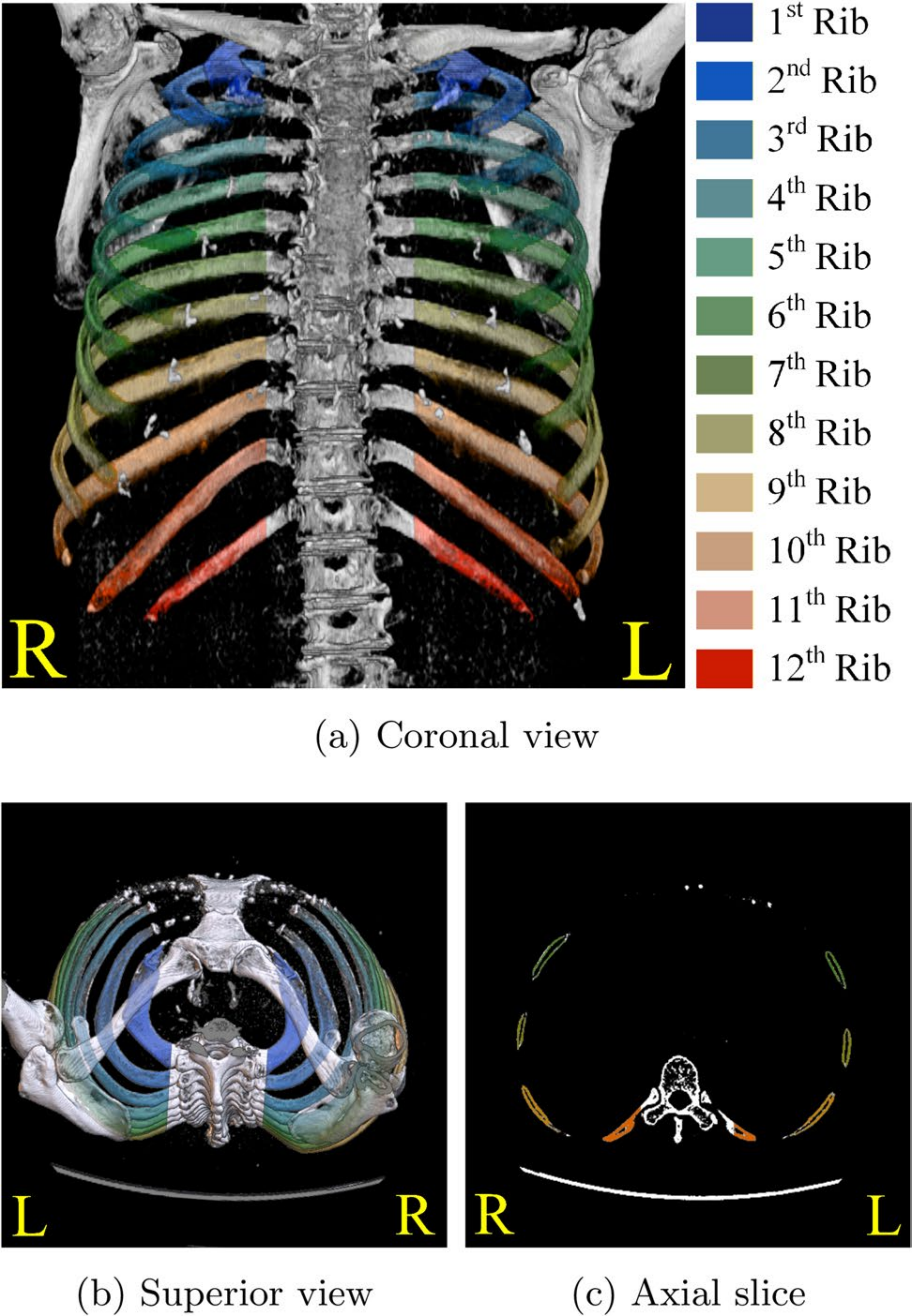
A sequential numbering result of the worst rib segmentation case by DCRS. Each rib is denoted with individual colors

The remarkable numbering accuracy and throughput are directly attributed to the robust rib segmentation results of the proposed DCRS, as demonstrated in Fig. 9. This case exhibits scoliosis, spine-sternum misalignment, and distorted patient position. DCRS successfully preserves 12 rib pairs for numbering Fig. 9a, although posterior rib segmentation is challenging due to anatomical complexity as shown in Fig. 9b. The axial slice Fig. 9c demonstrates that DCRS identifies 10th rib with posterior rib omission. This is because the kernel boundary in RONI removal includes not only spine, transverse process, and sternum but also posterior ribs. Nevertheless, the RONI removal prevents the DL model from misidentifying the transverse process with sagittal ribs, further facilitating the SNOFF algorithm to efficiently explore the connected rib voxels. There is no difficulty in numbering the rib sequence with the DCRS output, however, we further discuss the posterior rib omission for typical failure analysis in Section 4.

**Fig. 10:**
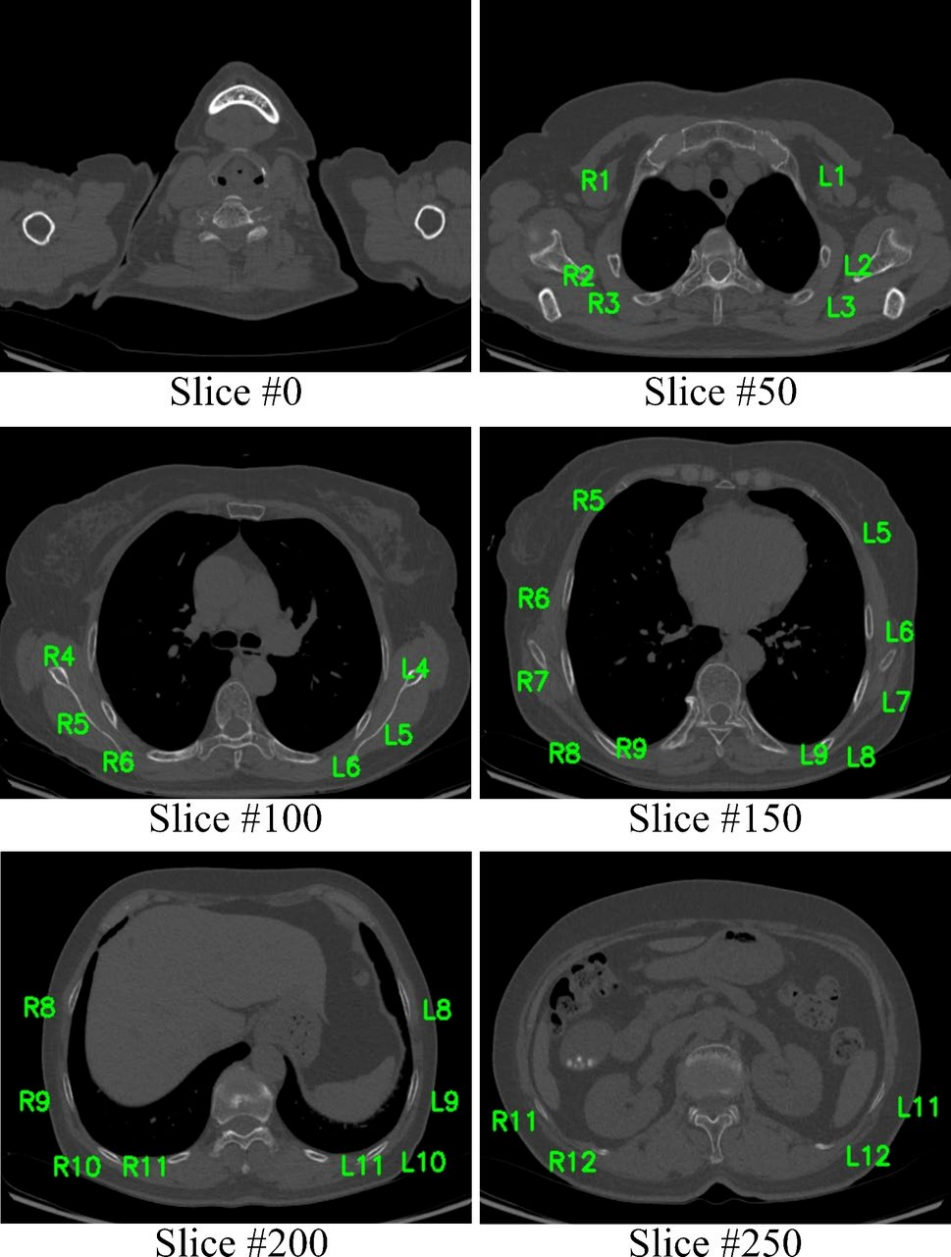
Consistent voxel-wise rib number and laterality annotations across the axial slice outputs from the proposed DCRS

Sequential rib numbering results are depicted in Fig. 10. Rib annotations are precisely represented regardless of whether the CT scan viewer follows radiological or neurological conventions, as they are embedded in all rib voxels. It is worth noting that the annotations are robust to various patient positions and anatomical deformities. The annotated chest CT from DCRS has multifaceted clinical relevance, enhancing diagnostic precision and clinical efficiency. Assured rib numbering enables radiologists to precisely localize rib fractures, tumors, or metastatic diseases, where the exact location significantly influences the treatment plan and prognosis. The robustness of rib annotations underscores the clinical utility of the DCRS under complex anatomical conditions (e.g., severe scoliosis, fractures, patient positioning variations). Moreover, high throughput translates into faster interpretation of chest CT. The throughput of proposed DCRS corresponds to 8.9 seconds per CT volume, requiring only an additional 0.5% of time into the chest CT scan processes which takes 30 min on average [33]. This speed expedites decision-making in clinical settings, reducing the time from patient presentation to diagnosis and subsequent treatment initiation.

## Discussion

The coarse-to-fine approach of the proposed DCRS significantly improves 3D rib construction reliability, however, DCRS outputs have posterior rib omissions in common due to RONI removal. As detailed in Section 2, patient positions and spinal deformities can broaden the pixel intensity range within the spinal region on the projected image. Spine highlighting integrates spinal components such as vertebra and transverse process. These components have low pixel intensities owing to their various positions. Median filter alters the low intensities to a comparatively high intensities and vice versa, enhancing a bit of pixel intensities which denote the ribs near the spine. The subsequent adaptive thresholding preserves these partial rib pixels and spine pixels together, treating these pixels as the spinal region. A personalized kernel eliminates this region from the CT volume. The removed region is excluded from 3D rib construction. This is because the SNOFF constructs 3D volume by extending seeds from sagittal rib predictions in posterior ribs towards anterior ribs. This segmentation strategy affects comparatively lower performances of the first ribs and twelfth ribs, as their posterior parts are removed.

**Fig. 11:**
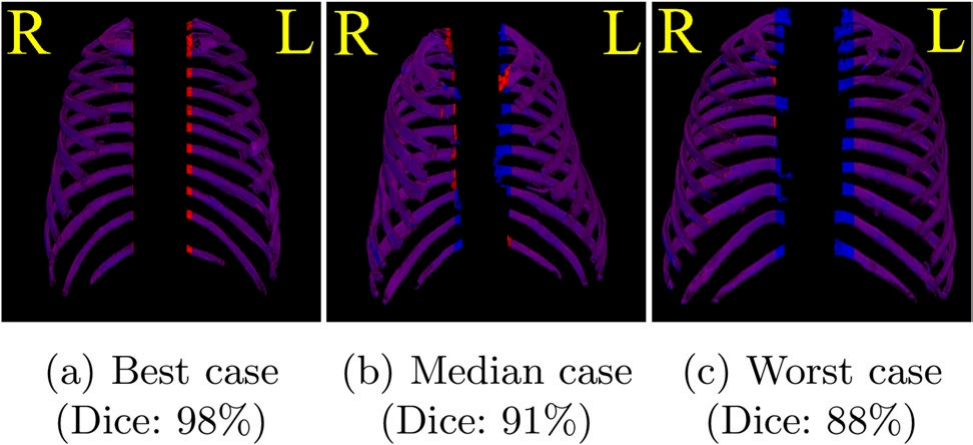
Overlays of DCRS rib predictions and corresponding ground truth labels by the highest, medium, and lowest Dice values. Purple area indicates the common voxels, whereas red and blue area refer to voxels only included in DCRS output and label, respectively

Quantitative and qualitative analyses in Section 3 indicate that the DCRS has a definite edge over other methods in the face of its drawback. Further analysis is necessary to verify that sequential rib numbering is robust against DCRS output. Figure 11 depicts three different DCRS outputs based on their Dice values, each overlapping with its corresponding label. The purple region represents a complete match between the output and the label. The red region refers to the actual rib voxels that are not included in the label. This erroneous labeling occurs since the segmentation labels are extracted by the rule-based algorithm [11, 14]. As shown in Fig. 11a and 11b, our proposed method effectively captures the red regions along with purple regions. This implies that the actual precision of the DCRS is likely to be higher than 92.43%. The blue region comprises rib voxels missed by the DCRS. These regions depict 5.27% of the missed rib voxels in 50 chest CT volumes. All the missed voxels are adjacent to the spine, as illustrated in Fig. 11b and 11c. This is because the DCRS eliminates the spinal region with kernel which has a slightly larger shape than the actual spine. It is worth noting that some red voxels also appear near the spine in the worst case.

**Table 3:** Segmentation performance for each rib index

Rib Index	Dice	IoU
1	78.79±22.09	70.05±22.00
	(72.50, 85.08)	(63.85, 76.25)
2	91.51±13.22	86.92±13.46
	(87.85, 95.17)	(83.34, 90.50)
3	94.94±7.32	91.37±8.83
	(92.90, 96.98)	(88.73, 94.01)
4	96.16±2.52	92.76±4.46
	(95.45, 96.87)	(91.54, 93.98)
5	96.50±2.25	93.27±4.10
	(95.88, 97.12)	(92.13, 94.41)
6	97.32±2.10	94.80±3.90
	(96.75, 97.89)	(93.68, 95.92)
7	**97.42±1.96**	**94.98±3.62**
	(96.89, 97.95)	(93.89, 96.07)
8	97.41±1.95	94.97±3.52
	(96.90, 97.92)	(93.92, 96.02)
9	97.33±2.11	94.89±3.80
	(96.75, 97.91)	(93.78, 96.00)
10	95.59±6.60	92.42±7.31
	(93.70, 97.48)	(90.31, 94.53)
11	94.57±3.72	89.91±6.12
	(93.47, 95.67)	(88.11, 91.71)
12	88.17±9.66	80.33±11.85
	(85.52, 90.82)	(76.91, 83.75)

Values are shown as mean±SD with 95% CI on the line below. The best and second best results are highlighted in **bold** and underline, respectively

We analyze the segmentation performance per rib index of the proposed DCRS to identify where it struggles, as shown in Table 3. A distinct trend across the rib sequence indicates the highest Dice and IoU observed at the seventh ribs and the lowest ones at the first ribs, corresponding to a performance gap of approximately Dice 18.6% and IoU 24.9%. The floating ribs achieve 11.9% and 14.7% higher Dice and IoU than first ribs, in the face of their short and thin structures. This robustness is attributed to the dual-sagittal coarse segmentation that provides overall rib structures, enabling the model to readily identify floating ribs. The proposed DCRS maintains high segmentation performance across all rib indices, particularly with average Dice 95.88% and IoU 92.63% among middle ribs (from second to eleventh). The comparatively lower performances of the first ribs and the twelfth ribs are due to RONI removal.

**Fig. 12:**
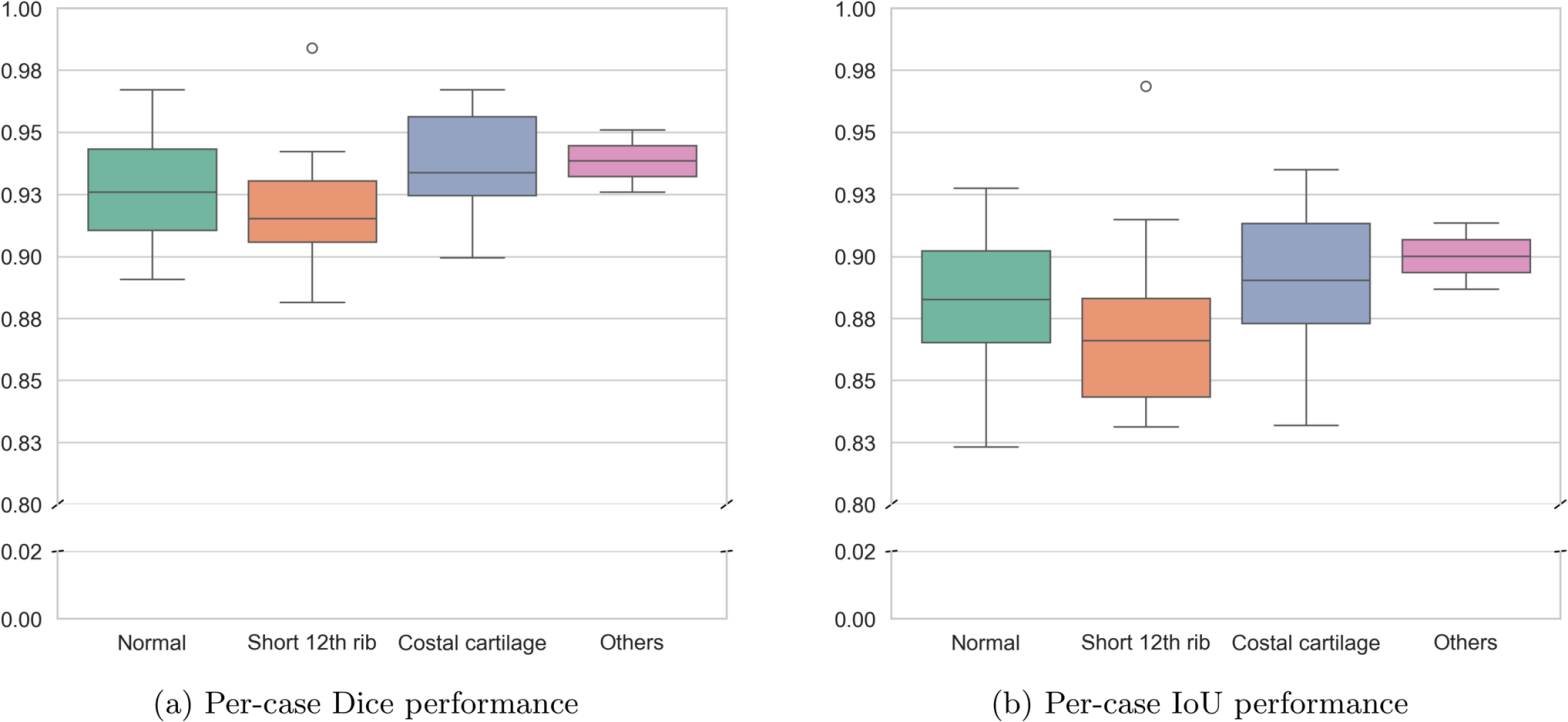
Per-case segmentation performance across anatomical groups. Each boxplot illustrates the distribution of per-case performance across groups, including normal, short 12th rib, costal cartilage, and others (fracture and scoliosis) cases

**Table 4:** 2D Rib segmentation performances over 7 different weight combinations of mixed loss

Weight Ratio	1:0	4:1	3:2	1:1	2:3	1:4	0:1
IoU	89.1	91.9	92.2	**93.1**	92.9	92.7	92.5
Accuracy	99.9	99.9	99.9	**99.9**	99.9	99.9	99.9
Loss	0.002	0.008	0.012	0.022	0.036	0.065	0.095

The highest IoU and accuracy are achieved with a weight ratio of 1:1, as emphasized in **bold**

**Fig. 13:**
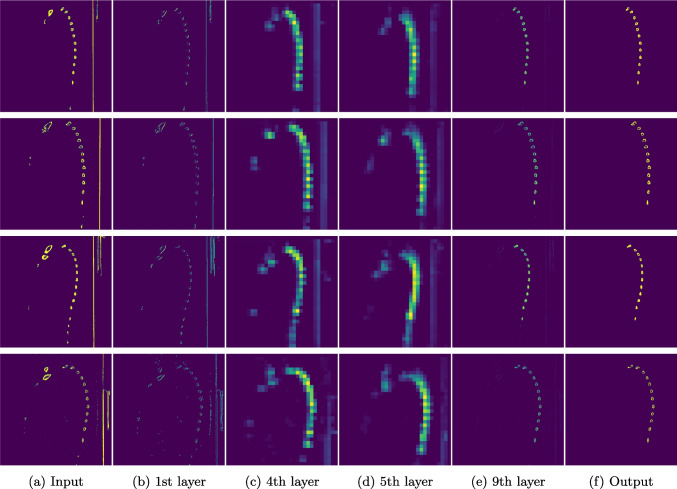
Feature map visualizations from the first and the last layers of encoder (1st and 4th layers) and decoder (5th and 9th layers). The colormap represents activation magnitude (higher activations in yellow and lower activations in blue). The model gradually suppresses background noise due to HU variations and unremoved structures (i.e., fragments of clavicle, sternum, and transverse process) during RONI removal, in accordance with model depth. The vertical rib patterns are enhanced as input features undergo decoder. Note that outputs are arbitrarily resized with the same shape for visibility

A per-case group-wise analysis evaluates the robustness of the proposed DCRS on anatomical variations, as illustrated in Fig. 12. The testset is divided into normal (23 cases), short twelfth rib (12 cases), costal cartilage (12 cases), and others (2 fracture and 1 scoliosis cases). DCRS maintains consistently high segmentation performance across all groups. The highest Dice and IoU are observed in the short 12th rib group, indicating that DCRS accurately segment even shortened ribs. The costal cartilage group also exhibits stable and strong performance despite its low-intensity and discontinuous bone structures. The “others” group achieves comparable results with narrow variability, demonstrating that DCRS generalizes robustly even to fractures and scoliosis.

**Ablation Study** The coarse 2D sagittal rib segmentation performance affects subsequent fine 3D rib segmentation and sequential numbering. Each weight of combination loss is crucial for DL model to capture miscellaneous shape of sagittal ribs while assuring identification of all ribs. We conduct seven different weight combinations of BCE with logits loss and IoU loss to set the best weight ratio for DL model training. The validation performance of each combination is shown in Table 4. The DL model shows the best segmentation performance when the equal ratio is assigned to each loss function, showcasing 99.9% accuracy and 93.1% IoU. The total loss is lowest when the model is trained only by the BCE with logits loss, however, the segmentation performance is 4.5% lower than the equally weighted combination loss. Equal weighting between BCE and IoU losses provides an optimal balance by simultaneously ensuring pixel-wise classification accuracy and morphological segmentation similarity. This study adopts equal weight for 2D sagittal rib segmentation to provide the robust and precise cornerstones for 3D rib construction.

The outstanding segmentation performance indicates that the coarse DL segmentation precisely captures sagittal ribs against the background, by learning entire spatial information of ribs within local regions of chest CT. We further analyze the segmentation process of the black-box model to mitigate concerns regarding its non-deterministic nature, as illustrated in Fig. 13. The four different samples are shown from top to bottom, in order of the noise includability. The pivot sagittal slices Fig. 13a still contain noises after preprocessing. Noises comprise not only the clavicle, fraction of 1st rib, and examination table which are not the target in the RONI removal stage, but also sternum and transverse process due to anatomical variations. The 1st feature maps Fig. 13b respond uniformly to all strong edges, outlining every object in the slice. The final activations of the contracting path Fig. 13c suppress non-rib textures and amplify the characteristic rounded profiles of the ribs, visible as a dotted vertical arc. The bottleneck layer outputs Fig. 13d coalesce into a smoother and more continuous band aligned with the vertical arrangement of ribs, while background structures are further attenuated. The feature maps become probability maps of rib presence Fig. 13e after passing through the expanding path and sigmoid function. Every single sagittal rib Fig. 13 is obtained by applying threshold of 0.5 to probability maps. These visualizations of inherent feature refinement process demonstrate the robustness of rib segmentation even with significantly decreased input data.

## Conclusion

This study presents Dual sagittal-guided Coarse-to-fine Rib Segmentation (DCRS), a framework for accurate 3D rib segmentation and sequential numbering in chest CT scans. The framework includes two main stages. The coarse 2D stage robustly identifies sagittal ribs across complex anatomies. The fine 3D stage constructs complete rib structures from the sagittal outputs using the SNOFF algorithm. Subsequent sequential rib numbering provides consistent reference points for chest CT interpretation, aiding in the precise localization of affected areas. DCRS achieved state-of-the-art segmentation Dice scores and demonstrated superior rib numbering accuracy and computational efficiency compared with previous approaches on the RibSeg and RibFrac datasets. The resulting segmentation and numbering pipeline enables accurate anatomical referencing and reduces repetitive cross-referencing in clinical workflows. Although DCRS performs strongly, additional validation on low-dose and trauma CT data is needed to confirm its robustness across diverse clinical conditions. Future work will focus on extending DCRS to multimodal learning, automated lesion detection, and enhanced interpretability, further advancing its use in intelligent chest imaging systems.
